# A new segmentation algorithm for measuring CBCT images of nasal airway: a pilot study

**DOI:** 10.7717/peerj.6246

**Published:** 2019-01-28

**Authors:** Chen Zhang, Robin Bruggink, Frank Baan, Ewald Bronkhorst, Thomas Maal, Hong He, Edwin M. Ongkosuwito

**Affiliations:** 1 The State Key Laboratory Breeding Base of Basic Science of Stomatology (Hubei-MOST) & Key Laboratory of Oral Biomedicine Ministry of Education, School & Hospital of Stomatology, Wuhan University, Wuhan, China; 2 Department of Dentistry, Section of Orthodontics and Craniofacial Biology, Radboud University Nijmegen Medical Center, Radboud University Nijmegen, Nijmegen, Netherlands; 3 3DLAB The Netherlands, Radboud University Medical Center, Radboud University Nijmegen, Nijmegen, Netherlands; 4 Department of Dentistry, Section of Preventive and Restorative Dentistry, Radboud University Nijmegen Medical Center, Radboud University Nijmegen, Nijmegen, Netherlands; 5 Department of Oral and Maxillofacial Surgery, Radboud University Nijmegen Medical Center, Radboud University Nijmegen, Nijmegen, Netherlands

**Keywords:** Image segmentation, Nasal airway, CBCT, 3D printing, Airway model

## Abstract

**Background:**

Three-dimensional (3D) modeling of the nasal airway space is becoming increasingly important for assessment in breathing disorders. Processing cone beam computed tomography (CBCT) scans of this region is complicated, however, by the intricate anatomy of the sinuses compared to the simpler nasopharynx. A gold standard for these measures also is lacking. Previous work has shown that software programs can vary in accuracy and reproducibility outcomes of these measurements. This study reports the reproducibility and accuracy of an algorithm, airway segmentor (AS), designed for nasal airway space analysis using a 3D printed anthropomorphic nasal airway model.

**Methods:**

To test reproducibility, two examiners independently used AS to edit and segment 10 nasal airway CBCT scans. The intra- and inter-examiner reproducibility of the nasal airway volume was evaluated using paired *t*-tests and intraclass correlation coefficients. For accuracy testing, the CBCT data for pairs of nasal cavities were 3D printed to form hollow shell models. The water-equivalent method was used to calculate the inner volume as the gold standard, and the models were then embedded into a dry human skull as a phantom and subjected to CBCT. AS, along with the software programs MIMICS 19.0 and INVIVO 5, was applied to calculate the inner volume of the models from the CBCT scan of the phantom. The accuracy was reported as a percentage of the gold standard.

**Results:**

The intra-examiner reproducibility was high, and the inter-examiner reproducibility was clinically acceptable. AS and MIMICS presented accurate volume calculations, while INVIVO 5 significantly overestimated the mockup of the nasal airway volume.

**Conclusion:**

With the aid of a 3D printing technique, the new algorithm AS was found to be a clinically reliable and accurate tool for the segmentation and reconstruction of the nasal airway space.

## Introduction

With emerging evidence of how an altered breathing pattern affects craniofacial development and causes sleep-related breathing problems, the structure of the upper airway is of growing interest to researchers (Santander, Sievers & Moser, 2016; Ucar & Uysal, 2012), among which nasal airway is a common site for obstruction (Mohan et al., 2018). Sufficient nasal airway size is crucial for normal nasal breathing pattern. Obstructions in nasal airway might lead to the shift from nose breather to mouth breather and the subsequent adverse outcome. The diagnosis and treatment of sleep-disordered breathing or an abnormal craniofacial growth pattern require reliable and accurate upper airway analysis (Graf et al., 2016; Park et al., 2016). Lateral cephalometry, as an essential tool for orthodontic assessment, had played a vital role in routine upper airway analysis (Armalaite & Lopatiene, 2016); however, lateral cephalometry presents only a three-dimensional (3D) structure in a two-dimensional manner, which dispenses with much useful information concerning airway morphology. With the emergence of 3D technology, computed tomography (CT) offers great advantages compared to conventional two-dimensional cephalometry for its ability to capture 3D information (Aboudara et al., 2009; Armalaite & Lopatiene, 2016).

Cone beam computed tomography (CBCT), known for its low radiation dosage compared to conventional CT (Loubele et al., 2009), is widely accepted for diagnosis in dental practice (Dawood, Patel & Brown, 2009). Clinicians prefer it for investigating hard and soft tissue at a lower radiation dose (Guijarro-Martinez & Swennen, 2011). The relatively recent involvement of dental practitioners with sleep-disordered breathing has furthered the application of CBCT in the field of airway assessment (Zimmerman, Lee & Pliska, 2017). After CBCT image capture, the digital imaging and communications in medicine (DICOM) files will be further processed by different software packages for segmentation, reconstruction, and calculation.

For airways, the most commonly used segmentation techniques were based on the principle of region growing with set boundaries (Pham, Xu & Prince, 2000). However, most of those commercial software packages offering this function were more or less a “black box,” in which the details of their algorithm were unknown to the users. Unlike a transparent algorithm with visible code, in a “black box,” users can only input scans and directly get the measurements they require. The step-by-step process of getting results, which might be vital, in case errors were detected, will then remain unclear. Numerous assessments are available to evaluate commercial software packages in airway segmentation, and some general pitfalls have been noted. First, although the nasal airway space plays an important role in the scope of breathing (Alsufyani et al., 2016), its intricate anatomy with the nasal septum surrounded by sinuses and nasal turbinates complicates the segmentation and reconstruction of an accurate 3D morphological model of the nasal airway space. Thus, most studies have bypassed assessment of the nasal cavity, and in the limited number of studies that have focused on it, researchers found that commercial software packages yield reliable results but poor accuracy (De Water et al., 2014; El & Palomo, 2010). Second, the lack of a gold standard for accuracy assessment has consistently hampered research (Alsufyani, Flores-Mir & Major, 2012), and the few studies of the nasal airway have been obliged to use manual segmentation results for this purpose (Alsufyani et al., 2016; Bui, Ong & Foong, 2015; De Water et al., 2014; El & Palomo, 2010). To obtain a true gold standard for accuracy, some researchers have begun using generic hollow models or phantoms instead of existing CT data (Ghoneima & Kula, 2013; Schendel & Hatcher, 2010; Weissheimer et al., 2012). Recently, two studies reported on a state-of-the-art 3D printing technique to produce anthropomorphic human skull and pharyngeal airway phantoms for comparing different CT scanners (Chen et al., 2017a) and various software packages (Chen et al., 2017b) when measuring the oropharyngeal airway. However, the nasal airway remained unexplored. To the best of our knowledge, no study has focused on the reproducibility and accuracy of any software packages in nasal airway segmentation using a lifelike anthropomorphic model.

For this sort of exploration, software is needed that can separate the nasal airway from its complex surrounding structures and precisely reconstruct and measure it. The aim of this pilot study was to establish an algorithm for nasal airway space analysis and appraise its reproducibility and accuracy using a 3D printed anthropomorphic nasal airway model.

## Material and Methods

To test the candidate algorithm, we randomly selected the nasal airway CBCT data files for five patients from the orthodontic clinic database of the School of Dentistry, Radboud University Nijmegen, the Netherlands. The data were collected using an i-CAT 3D Imaging System (Imaging Sciences International Inc, Hatfield, PA, USA) under the protocol of 120 KV(p), five mA, 7 s exposure time, and 0.4 mm voxel size. Those CBCT images were taken for specific diagnostic purposes, such as the presence of complex impacted teeth or need for orthognathic surgery. All procedures performed in studies involving human participants were in accordance with the ethical standards of the institutional and/or national research committee and with the 1964 Helsinki Declaration and its later amendments or comparable ethical standards. Ethical approval was obtained from the local ethics committee of Radboud University Nijmegen (2018-4112).

**Figure 1:**
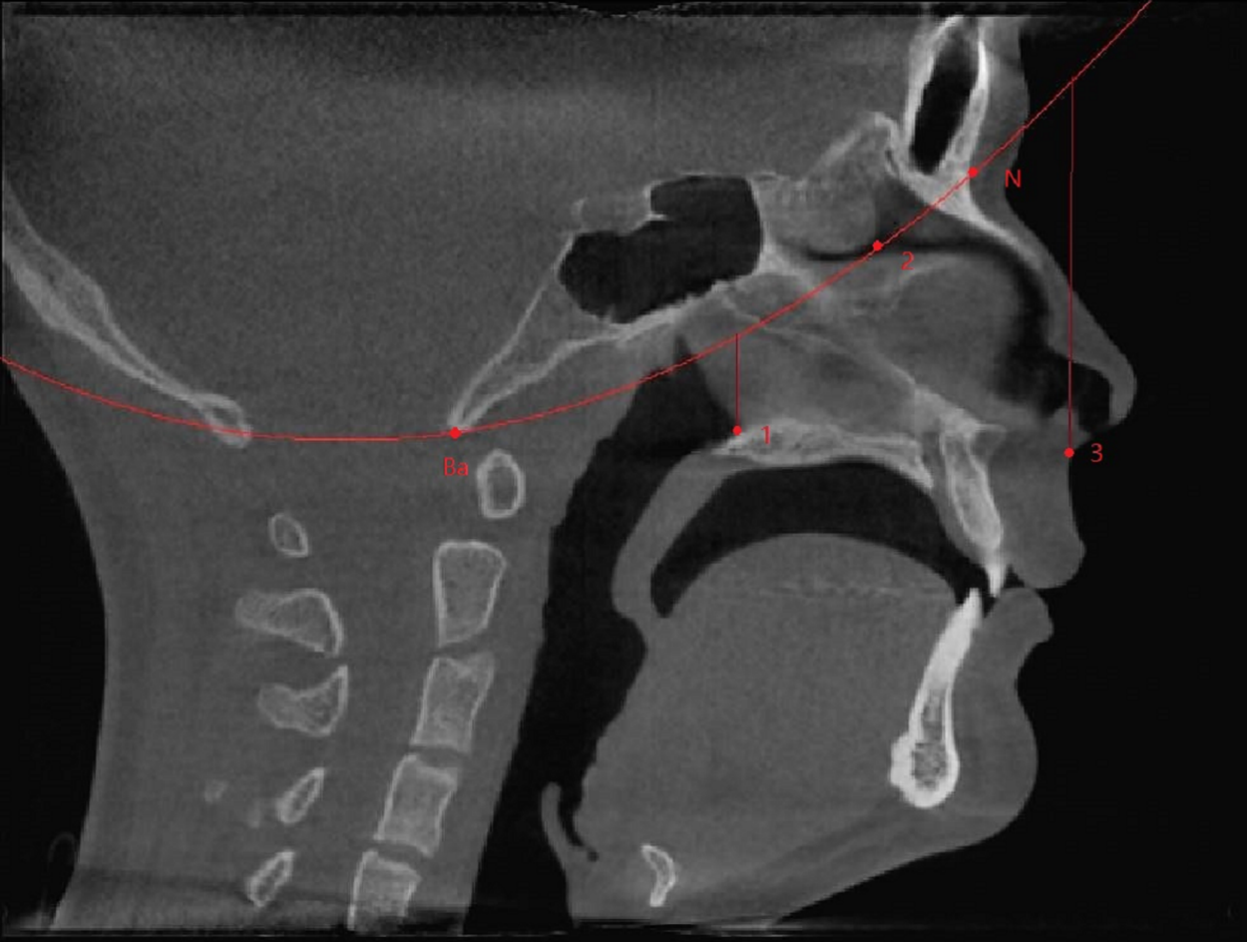
Region of interest. Ba, basion; N, nasion; (1) the most anterior point of the posterior border of the nasal septum; (2) the most posterior-inferior point of ethmoid sinus; (3) the most posterior point of the anterior nasal nares; Anterior border: the vertical plane passing point 3; Posterior border: the vertical plane passing point 1; Superior border: A spline curve by basion (Ba), point 2, and nasion (N); Inferior border: natural nasal cavity floor.

The region of interest (ROI) of the nasal airway defined in this study was limited to the nasal cavity: the anterior border was the vertical plane passing the most posterior point of the nasal nares, and the posterior border was the vertical plane passing the most anterior point of the posterior border of the nasal septum. A spline curve defined by basion (Ba), the most posterior-inferior point of the ethmoid sinuses, and the nasion (N) indicated the superior border, leaving the palate as the natural inferior border of the nasal airway space (Fig. 1). Thus, the left and right nasal airway spaces were separated by the septum.

**Figure 2:**
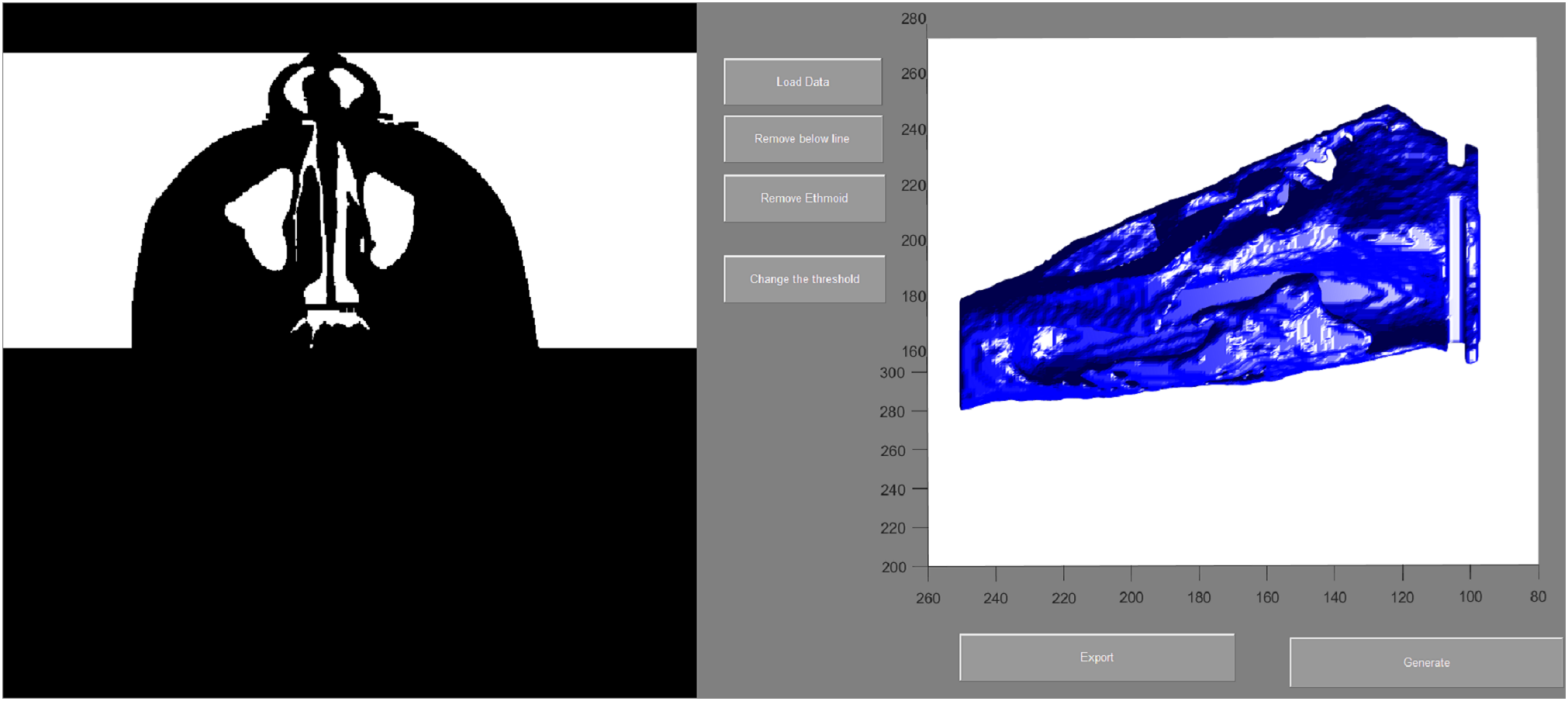
User interface of airway segmentor when segmenting nasal airway.

To evaluate the airway volume in CBCT images, a custom-made software program developed with MATLAB software (MATLAB 2017a; The MathWorks, Inc., Natick, MA, USA) was used for semi-automatic segmentation and analysis. This tool, designated as the airway segmentor (AS), edited and reconstructed the ROI of the nasal airway in five major steps. The first step was setting a binary threshold on the DICOM images for discrimination between tissue and air. Second, the user determined the ROI based on the abovementioned predefined references. The third step was to prepare the image set by smoothing (to reduce the noise in the data, this can affect the region growing as high intensity voxels could not be included) using median filter and Gaussian and then setting boundaries by closing the connections between any paranasal sinuses seen within the ROI and the nasal airway. This step was achieved by manually “blocking” those connections from the binary images with the use of an innovative and user-friendly interface. Fourth, the maxillary sinuses and other non-connected parts were removed, and only the nasal space of interest was reconstructed using region growing algorithm. Finally, the resulting volume was extracted and could be further corrected according to the voxel sizes, resulting in a volume measured in mm^3^. An example of the resulting nasal airway was visualized in Fig. 2.

**Figure 3:**
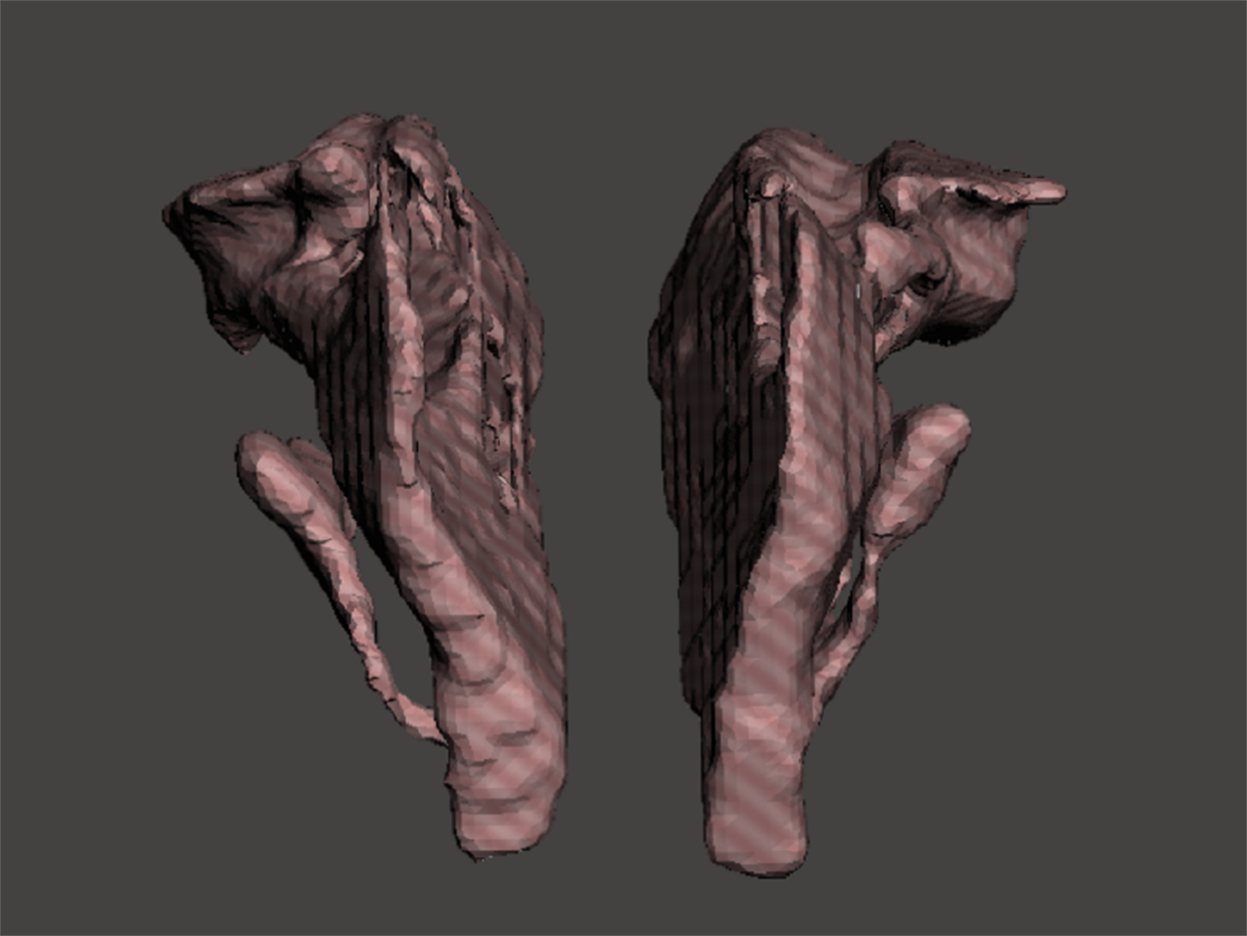
STL files of the reconstructed nasal airway model.

**Figure 4:**
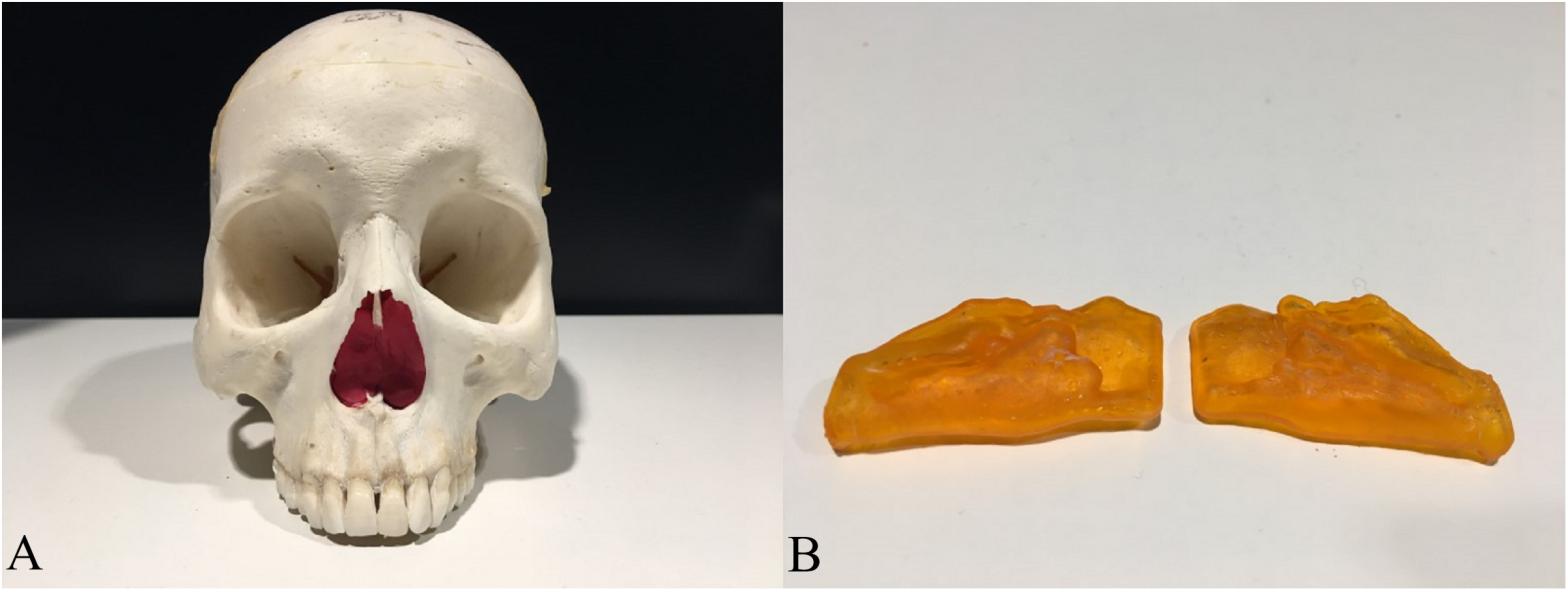
The human skull and the 3D printed hollow nasal airway model embedded in with playdough. (A) The skull with airway model and playdough inside; (B) the printed airway model.

To establish a measuring object to support a gold standard and offer lifelike morphology, 3D printing technology was used. A 3D model containing one pair of nasal airway spaces from a 16-year-old male patient was generated by AS and exported as a Standard Tessellation Language (STL) file (Fig. 3). The STL file was scaled down in Autodesk Meshmixer (Autodesk Inc., San Rafael, CA, USA) before printing to be fitted in the skull. The hollow airway space model was then 3D printed according to the STL file by the atum3D DLP (Digital Lighting Processing) station V2.0 (atum3D, Gouda, the Netherlands) using a DLP technique, with a soft tissue mimic resin (3DM-ABS; 3D-Materials, Feldkirch, France). By filling the hollow shell with water and measuring the added weight, an accurate measure of the inner volume could be calculated because the density of water is constant. Wrapped in Play-Doh (Kids Creative clay set; Tollkühn Shoppartner GmbH, Stuhr, Germany), the airway model was then embedded into a dry human skull in the anatomical position. In this way, a phantom comprising a pair of hollow nasal airway spaces of precise volume and lifelike morphology, a dry skull, and Play-Doh acting as the soft tissue equivalent was made (Fig. 4). The phantom was scanned by CBCT under the same conditions as described above and a DICOM file generated. The volume of the airway model acquired physically was later used as the gold standard to test accuracy.

For appraisal of this algorithm, the reproducibility and accuracy were assessed. The reproducibility was defined as the agreement between the measurements made by the same examiner (intra-examiner) or made by different examiners independently (inter-examiner). Accuracy was the agreement between the measurements reported by the algorithm/software and the gold standard.

### Reproducibility test

Briefly, 10 nasal airway scans from five individuals were edited and segmented by two examiners independently to test the reproducibility.

A segmentation algorithm focusing on the nasal airway, unlike other software packages dealing with the tube-like pharyngeal airway only, must be able to edit the DICOM file to separate the nasal airway from the connected sinuses and then to delete sinuses. Therefore, the reproducibility of nasal airway processing is more challenging than with processing the pharyngeal airway.

Using AS, altogether 10 nasal airway spaces from the five patients were edited and the volume calculated twice by examiner #1, with a 10-day interval, and once by examiner #2. For examiner #1, CBCT datasets were shuffled during the second examination to minimize recall bias. Examiner #2 conducted the whole process independently. Intra-examiner reproducibility was defined as the agreement between the two measurements of examiner #1, while inter-examiner reproducibility was defined as the agreement between the first measurement of examiner #1 and that of examiner #2.

### Accuracy test

Briefly, one pair of post-editing nasal airway was printed and measured by one examiner using different software.

**Figure 5:**
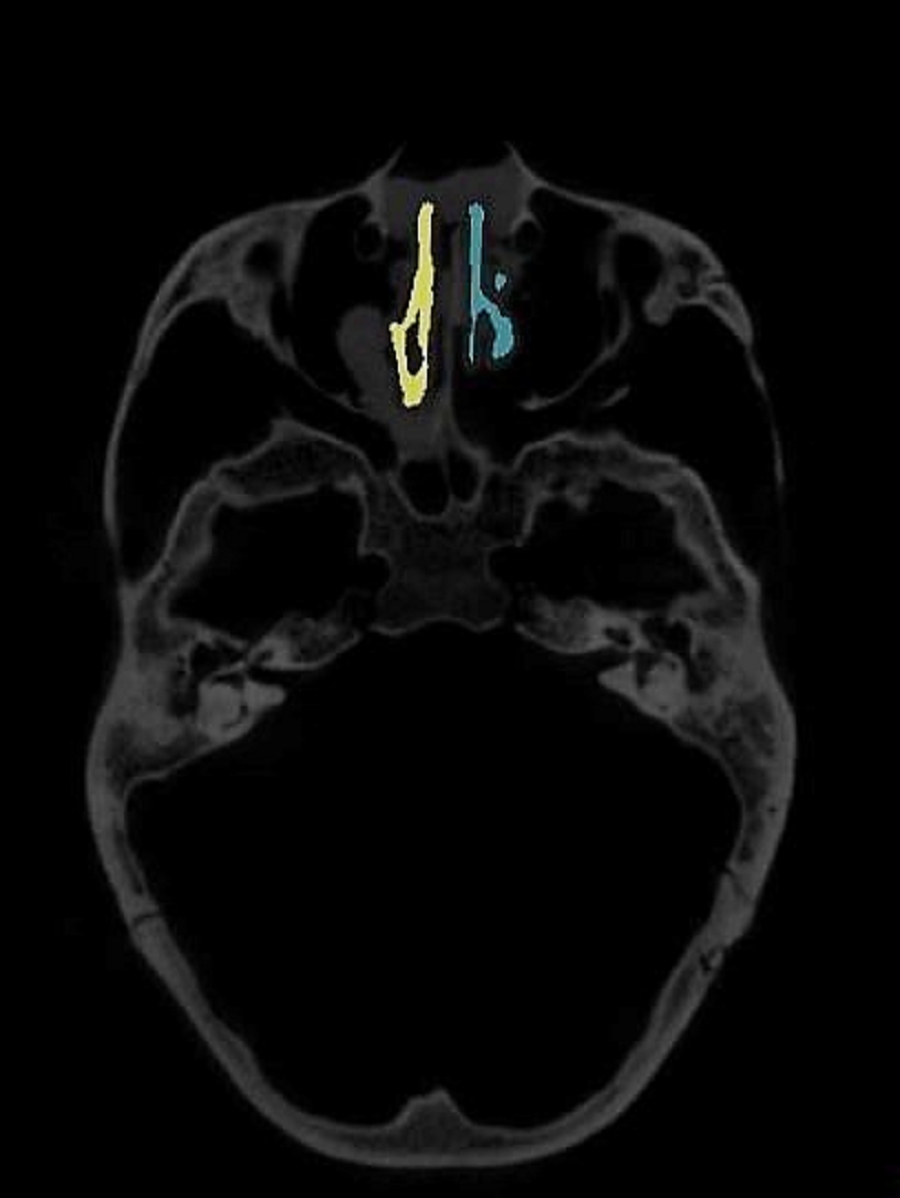
Segmentation of nasal airway space by MIMICS 19.0.

**Figure 6:**
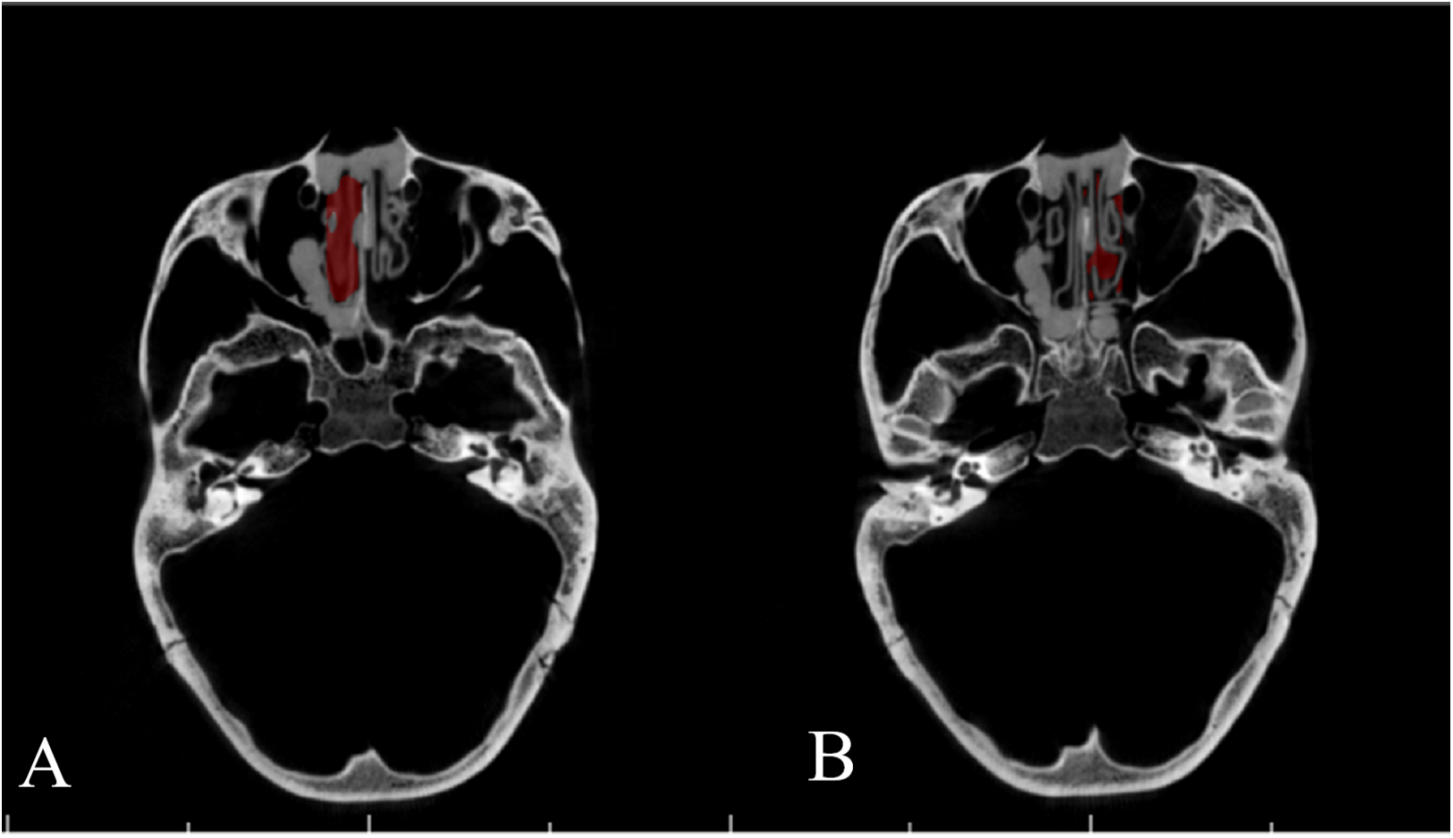
Segmentation of nasal airway space by INVIVO 5. (A) Right side; (B) left side.

Airway Segmentor and two other commercial software packages, MIMICS 19.0 (Materialise, Leuven, Belgium) and INVIVO 5 (Anatomage, San Jose, CA, USA), were used for calculating the inner volume of the pair of printed models (Figs. 5 and 6), according to their instruction manuals. Results then were compared to the gold standard. The threshold used for all three software packages was defined as the highest threshold value that retained the inner space of the resin model disconnected from the outer space.

### Statistical methods

To analyze measurement error, a paired *t*-test was applied to assess the structural differences between both intra- and inter-examiner measurements (Bartlett & Frost, 2008). Statistical significance was set at *P* < 0.05, which indicates a structural difference. The random error of the difference between two measurements was calculated as (standard deviation of the differences between two measurements)/√2. Reliability was calculated as the intraclass correlation coefficients (ICC), with a value >0.75 considered a good correlation. The Statistical Package for Social Sciences for Windows 24 (SPSS 24; IBM Corp., New York, NY, USA) was used for the analysis.

For accuracy, results from the different software packages, as well as the percentage of the gold standard value, were reported. Since, for a given post-editing scan, one software package would always return the exact same volume measurement, there was no standard deviation to report.

## Results

**Figure 7:**
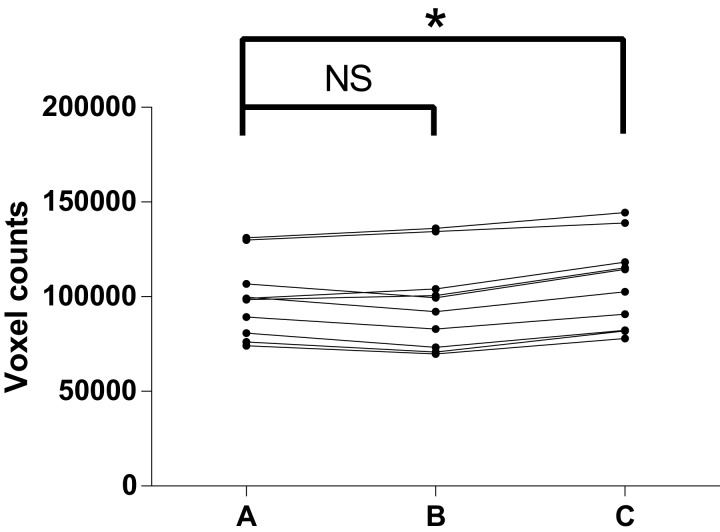
Test of reproducibility. (A) 1st measurement by C.Z; (B) 2nd measurement by C.Z; (C) measurement by R.B; NS, no significance; *P-value < 0.05.

In the test of reproducibility, descriptive data for the airway volume readings are listed in Table 1. As shown in Table 2 and Fig. 7, paired *t*-tests indicated no structural difference between the two readings from the same examiner, which together with the high ICC revealed a high intra-examiner reproducibility. A structural difference was seen in the inter-examiner reproducibility test, however. The random error of the difference of the readings between inter-examiner measurements was calculated to be 4,518.4, equivalent to 289.2 mm^3^, given the voxel size of 0.4 mm.

**Table 1:** Descriptive data of airway volume measurements.

	Samples	Means (voxel counts)	Std. deviation
1st measurement by examiner #1	10	98,516	20,020
2nd measurement by examiner #1	10	96,346	24,062
Measurement by examiner #2	10	106,709	23,662

**Note:**

The voxel counts = the real volume × 15.625, given the voxel is 0.4 × 0.4 × 0.4 mm.

**Table 2:** Intra-examiner (1st measurement by C.Z vs 2nd measurement by C.Z) and inter-examiner reproducibility (1st measurement by C.Z vs measurement by R.B) by paired *t*-test.

	Intraclass correlation coefficient	Random error (voxel counts)	Mean difference (voxel counts)	95% confidence interval of the difference		Significance (two-tailed)
				Lower	Upper	
Intra-	0.966	3,993.8	2,169.1	−1,870.8	6,209.0	0.255
Inter-	0.899	4,518.4	−8,193.9	−12,764.3	−3,623.5	0.003

**Note:**

The voxel counts = the real volume × 15.625, given the voxel is 0.4 × 0.4 × 0.4 mm.

In the present study, CBCT images of the phantom showed clear distinctions between bone, Play-Doh, and air in most of the area. Similar to the image from living patients, the grayscale for cortical bone in the images was around 800, while the grayscale of the Play-Doh was around 300 and the air around −1,000 (data not shown). AS reported 103.7% of the gold standard on the left side and 107.2% of the gold standard on the right side, while MIMICS reported results almost identical to those of AS. INVIVO 5 reported 131.1% of the gold standard on the left side and 144.6% on the right side (Table 3).

**Table 3:** Accuracy of different software compared to the gold standard (GS).

	GS	AS	MIMICS	INVIVO
Left side	1,220 mm^3^	1,265 mm^3^ (103.7% of GS)	1,268 mm^3^ (103.9% of GS)	1,600 mm^3^ (131.1% of GS)
Right side	830 mm^3^	890 mm^3^ (107.2% of GS)	893 mm^3^ (107.6% of GS)	1,200 mm^3^ (144.6% of GS)

## Discussion

The function of AS to edit and calculate nasal airway values was tested in this study, in which the reproducibility test focused mainly on the editing function and the accuracy test on the segmenting and calculating function. The segmentation time for a single nasal airway space ranged from 10 to 20 min, which was obviously shorter than previously reported manual segmentation (up to 3.5 h), similar to another semiautomatic segmentation algorithm (49 min for two sides) but longer than commercial software packages (5 min) (Alsufyani et al., 2016; El & Palomo, 2010; Seo et al., 2010; Tingelhoff et al., 2007). Noted that some of the previous work only segmented the nasal airway and the paranasal sinuses as a whole, and did not try to separate sinuses from the passage as we did.

Unlike the relatively simple tube-like pharyngeal airway, the nasal airway space has a complex anatomy surrounded by paranasal sinuses. The ideal software for segmenting the nasal airway space must be able to edit DICOM data to separate airway passages from sinuses. The borders between sinuses and the nasal airway are occasionally vague, and their identification can vary and from examiner to examiner, so it might be difficult to precisely replicate previous editing procedures. Thus, any editing process requires a critical appraisal of reproducibility. However, the results of this study indicated good intra-examiner reproducibility. Although the ICC value was high, the inter-examiner reproducibility was not as good because a structural difference was detected. The main possible reason for the inconsistency between examiners was that connections between paranasal sinuses show considerable variation with no clear landmarks, especially for sphenoidal, frontal, and ethmoid sinuses. Discarding those sinuses at the top of the nasal airway was done by drawing a spline curve passing the most posterior-inferior point of the ethmoid sinuses, which was sometimes hard to precisely locate for a given dataset. However, the statistically significant inter-examiner difference could be considered clinically insignificant (Lal et al., 2006) because the random error was less than 0.3 cm^3^. Further research to improve methods for separating the paranasal sinuses and air cells might increase the reproducibility of nasal airway segmentation.

The accuracy of AS and MIMICS was almost identical and clinically excellent while INVIVO 5 showed a substantially lower accuracy, with greatly overestimated volumes (Table 3). The reason for this inconsistency might trace to different segmentation methods (Pham, Xu & Prince, 2000). AS and MIMICS use a manual region growing method, which works well in objects with highly complex or even distorted morphology but still having highly contrasted grayscale readings for the adjacent structures. INVIVO 5, on the other hand, uses either automatic seed determination during regional growing or a method other than region growing because there is no manual “seed placing” action needed. Thus, it apparently caused more overflowed segmentation (Fig. 6) compared to MIMICS (Fig. 5).

Unlike previous studies in which the measurement error of the nasopharyngeal airway volume ranged from −31% to +15% (De Water et al., 2014; El & Palomo, 2010), all three software packages used in this study overestimated the volume, and AS reported results with measurement errors as low as +3.7% and +7.2%. The measurement error could be explained by partial-volume effects (Baumgaertel et al., 2009; Pham, Xu & Prince, 2000). Voxels right at the air–tissue border were either rendered as “tissue” or “air” during the segmentation process, depending on the threshold value. Therefore, a higher threshold value induced smaller tissue volume and larger air volume, and vice versa. Meanwhile, the threshold value for airway defined in this study was the highest value that kept the inner space of the resin shell from connecting with the outer space. For this reason, some voxels at the air–resin border could have been wrongly allocated to “air,” leading to overestimation.

Most of the previous airway studies have de facto used manual segmentation results or different imaging modalities as the gold standard in the absence of validation. Compared to acoustic reflection results, Dolphin 3D Imaging program was accurate in measuring anterior nasal airway but inaccurate in measuring total nasal airway (Tsolakis et al., 2016). Studies using generic hollow models instead of manual segmentation of the existing CT data (Ghoneima & Kula, 2013; Schendel & Hatcher, 2010; Weissheimer et al., 2012) have been carried out to overcome the lack of a gold standard and according to the generic model, CT scans with varies software were reliable and accurate. However, it is reasonable to suspect that these generic models are too simple in 3D detail to imitate real-world airway morphology, considering the complexity of the airway and tissues surrounding it. The choice to 3D print an anthropomorphic human skull and pharyngeal airway phantoms seemed to be an excellent way to mimic morphology when measuring the pharyngeal airway, as some groups have done (Chen et al., 2017a, 2017b). They found out that compared to the actual volume of the printed pharyngeal model, software readings from CT scan were reliable with underestimation. Because the nasal cavity is full of thin bony structures, though, current 3D printing techniques might not replicate every tiny detail within the nasal cavity. For this reason, we applied a dry human skull as the best substitute for living tissue.

One limitation of this study was that all of the CBCT datasets were acquired using one CBCT machine with the same image acquisition protocol. Because there are multiple CT scanners and image acquisition protocols, future studies may need to incorporate different machines and protocols to test the reproducibility and accuracy of various software packages. Moreover, the segmentation time for a single nasal airway space ranged from 10 to 20 min, which was still longer than the reported time needed of some commercial software packages for similar task. If anterior nares region and the small part of the nasal airway at the top need to be included, the operating time would be even longer. Improved coding and user interface are required for better efficiency. Also, only a small number of samples were used as the objects for measuring, studies with larger sample size would also be more convincing.

## Conclusion

The algorithm AS showed a good intra-examiner reproducibility and clinically acceptable inter-examiner reproducibility during the editing process of the nasal airway space. Furthermore, AS could precisely segment and calculate nasal cavity volume.

## Supplemental Information

10.7717/peerj.6246/supp-1 Supplemental Information 1 Raw data for reliability test.

10.7717/peerj.6246/supp-2 Supplemental Information 2 Reliability test from SPSS.

10.7717/peerj.6246/supp-3 Supplemental Information 3 Matlab code.

## Additional Information and Declarations

### Data Availability

The following information was supplied regarding data availability: The raw measurements are available in the Supplementary Files.
