# Supplementary material for: A new segmentation algorithm for measuring CBCT images of nasal airway: a pilot study
Source: PeerJ. 2019 Jan 28;7:e6246. doi: 10.7717/peerj.6246 (PMC6354662; doi:10.7717/peerj.6246)
Supplement: Supplemental Information 1 [file peerj-07-6246-s001.docx]

# Reliability test

First measurement by cz

74151

76113

106784

89252

99117

98356

80761

99719

131015

129887

Second measurement by cz

69745

70812

99389

82903

104112

100653

73247

92075

136077

134451

measurement by robin

77865

81989

114463

90809

118316

115302

82329

102627

144433

138961

# Accuracy test

Golden standard

| weight before injection(g) | | |
| --- | --- | --- |
| left | 3.04 |  |
| right | 3.27 |  |
|  |  |  |
| weight after injection(g) | | |
| left | 4.26 |  |
| right | 4.10 |  |
|  |  |  |
| water volume(mm^3^) | |  |
| left | 1220 |  |
| right | 830 |  |

Measured from ct: (under threshold :-380HU, which is the highest threshold that keeps the model inside space disconnected with the outer space)

Left: 1265.024mm^3^ (103.7% of the golden standard)

right: 889.92mm^3^ (107.2% of the golden standard)

| Results from invivo（mm^3^） | |
| --- | --- |
| left | 1600 |
| right | 1200 |

| Result from mimics （mm^3^） | |
| --- | --- |
| left | 1268 |
| right | 893 |
