# Supplementary material for: A new segmentation algorithm for measuring CBCT images of nasal airway: a pilot study
Source: PeerJ. 2019 Jan 28;7:e6246. doi: 10.7717/peerj.6246 (PMC6354662; doi:10.7717/peerj.6246)
Supplement: Supplemental Information 2 [file peerj-07-6246-s002.docx]

**T-Test**

[DataSet1] C:\Users\m\Desktop\phantom\phantom.sav

| **Paired Samples Statistics** | | | | | |
| --- | --- | --- | --- | --- | --- |
|  | | Mean | N | Std. Deviation | Std. Error Mean |
| Pair 1 | czmeasurement1 | 98515.5000 | 10 | 20019.78973 | 6330.81338 |
|  | czmeasurement2 | 96346.4000 | 10 | 24061.91570 | 7609.04585 |
| Pair 2 | czmeasurement1 | 98515.5000 | 10 | 20019.78973 | 6330.81338 |
|  | robinmeasurement | 106709.4000 | 10 | 23661.71345 | 7482.49079 |

| **Paired Samples Correlations** | | | | |
| --- | --- | --- | --- | --- |
|  | | N | Correlation | Sig. |
| Pair 1 | czmeasurement1 & czmeasurement2 | 10 | .984 | .000 |
| Pair 2 | czmeasurement1 & robinmeasurement | 10 | .971 | .000 |

| **Paired Samples Test** | | | | | | | | | |
| --- | --- | --- | --- | --- | --- | --- | --- | --- | --- |
|  | | Paired Differences | | | | | t | df | Sig. (2-tailed) |
|  |  | Mean | Std. Deviation | Std. Error Mean | 95% Confidence Interval of the Difference | |  |  |  |
|  |  |  |  |  | Lower | Upper |  |  |  |
| Pair 1 | czmeasurement1 - czmeasurement2 | 2169.10000 | 5647.32406 | 1785.84067 | -1870.75226 | 6208.95226 | 1.215 | 9 | .255 |
| Pair 2 | czmeasurement1 - robinmeasurement | -8193.90000 | 6389.02092 | 2020.38581 | -12764.33024 | -3623.46976 | -4.056 | 9 | .003 |
